# Gender Differences in the Longitudinal Relationship Between Psychological Distress and Perceived Social Support

**DOI:** 10.1111/aphw.70148

**Published:** 2026-04-29

**Authors:** Kieren J. Lilly, Natalia M. Simionato, Fiona Kate Barlow, Danny Osborne, Caroline Salom, Chris G. Sibley

**Affiliations:** ^1^ Institute for Social Science Research University of Queensland Brisbane Australia; ^2^ School of Psychology University of Auckland Auckland New Zealand; ^3^ School of Psychology University of Queensland Brisbane Australia

**Keywords:** cross‐lagged panel model, gender, mental health, psychological distress, social support

## Abstract

The benefits of social connections for well‐being are often assumed, yet few studies test whether social support predicts mental health over extended periods nor whether these effects are experienced equally across women and men. We address this gap by examining gender differences in the longitudinal associations between perceived social support and psychological distress using 14 annual waves of a nationwide panel study (*N* = 75,404; 62.1% women). Multigroup random intercept cross‐lagged panel modelling revealed that within‐person increases in perceived social support predicted subsequent declines in psychological distress over time for both women and men. However, this relationship was bidirectional, and the negative within‐person effects of psychological distress on perceived social support were *stronger* than the effects of perceived social support on psychological distress among men (but not women). Analyses exploring gender interactions with age, sexual identity and ethnicity revealed that the effects of psychological distress were strongest among older, New Zealand European and heterosexual men. These results suggest that men's psychological distress leads to decreased perceptions of social support from others in their lives, and this effect is more pronounced than the effect of social support over the same timeframe. The implications for health interventions are discussed.

Again and again a man would tell me about early childhood feelings of emotional exuberance, of unrepressed joy, of feeling connected to life and to other people, and then a rupture happened, a disconnect, and that feeling of being loved, of being embraced, was gone … Sadly, tragically, these men in great numbers were remembering a primal moment of heartbreak and heartache: the moment that they were compelled to give up their right to feel, to love, in order to take their place as patriarchal men.

– hooks (2003, p. 35)

Concerns about rising rates of loneliness among men have increased scholarly discussions about the importance of social connectedness (e.g. Nordin et al., 2024; van Rossum et al., 2025). Indeed, social connections are fundamental for good health; people with strong social connections report fewer symptoms of depression (Cruwys et al., 2014), anxiety (Wakefield et al., 2022) and post‐traumatic stress (Jones et al., 2012). People with greater social support also report greater psychological well‐being (e.g. Haslam et al., 2016; Kearns et al., 2017) and live longer (Holt‐Lunstad et al., 2015) than people with limited social support. Moreover, the effects of social connectedness on outcomes such as mortality are often *stronger* than behavioural risk factors (including substance use and poor physical fitness; see Holt‐Lunstad et al., 2015; Steffens et al., 2016). These findings suggest that social support and integration act as a ‘social cure’ (see Haslam, Jetten, et al., 2018; Jetten et al., 2012), improving health and well‐being among individuals facing various stressors.

Although there is a general consensus that social connections improve health and well‐being, poor health can also undermine social connectedness. For instance, Billedo et al. (2019) identified a negative bidirectional relationship between perceived social support and depression symptoms among international students. Research using clinical samples also suggests that perceived social support has negative reciprocal relationships with depression and posttraumatic symptoms among survivors of abuse (Steine et al., 2020), as well as psychological distress among people with chronic physical conditions (Debnar et al., 2024). Beyond clinical and convenience samples, studies using representative data from the United Kingdom (Yu et al., 2015), the Netherlands (Domènech‐Abella et al., 2021), New Zealand (Saeri et al., 2018) and Japan (Yamaguchi et al., 2023) also demonstrate a reciprocal relationship between social connectedness and mental health among both youth and adults (though the effects of social connectedness are generally stronger; see Saeri et al., 2018). Thus, the relationship between social connectedness and well‐being is complex, and *ill*‐being can undermine social connectedness over time.

Despite providing a reliable buffer for threats to well‐being, not all ‘social cures’ are created equal, and some groups reap fewer benefits than others from social support. For example, hooks (2003) highlights in our opening epigraph that group norms among men may promote unhealthy norms about social connectedness (a ‘social curse’; for discussion, see Jetten et al., 2017). Moreover, because mental health stigma undermines people's willingness to seek support (e.g. Staiger et al., 2020), different *help‐seeking* norms may impact the adverse effects of ill‐being on social connectedness. Thus, the relationship(s) between social connectedness and well‐being may differ across groups. In the current study, we test this possibility by examining the longitudinal relationships between perceived social support and psychological distress among a nationwide panel sample of women and men. We focus on women and men as two interrelated groups with differing traditional norms surrounding social connectedness and mental health (for discussion, see van Rossum et al., 2025). Specifically, we argue that traditional gender norms affect how people experience social support *and* how they respond to psychological distress (e.g. Mostoller & Mickelson, 2024; Nam et al., 2010).

## GENDER DIFFERENCES IN THE BENEFITS OF SOCIAL SUPPORT

Traditional gender norms encourage women to create emotional bonds with others (see Barbee et al., 1993). Accordingly, women tend to have larger social networks (Antonucci & Akiyama, 1987) and hold more positive attitudes towards help‐seeking than men (Nam et al., 2010). Conversely, support‐seeking is generally discouraged among men (Galdas et al., 2005; Mostoller & Mickelson, 2024). As a result, men tend to place lower value on and *expect* less social support from friends compared to women (Williams et al., 2022). They also show less desire to disclose negative information to others (Carbone et al., 2024) and underestimate the importance of social factors like support for health (Haslam, McMahon, et al., 2018). These differences suggest that social connectedness may be a more readily available and effective buffer of distress among women compared to men (see Barbee et al., 1993). Supporting this possibility, prior cross‐sectional research suggests that social support is more strongly associated with mental health among young women (compared to young men; Johansen et al., 2021), and women benefit from family and work‐based support to a greater extent than men (Perrewé & Carlson, 2002). Although longitudinal research is limited, one Australian longitudinal study found that increased social support was associated with greater improvements to mental health for young people and women (compared to older people and men, respectively; Milner et al., 2016). Collectively, these studies suggest that women have greater access to social support *and* gain more benefits from increased social support than men.

## GENDER DIFFERENCES IN RESPONSES TO PSYCHOLOGICAL DISTRESS

In addition to the likely asymmetrical benefits of social support, there are reasons to expect gender differences in the effects of distress on perceived social support. Because traditional gender roles position men as self‐reliant and stoic, experiencing mental ill health is seen as incongruent with masculinity. Indeed, ill mental health among men is shrouded in shame and stigma (e.g. see Staiger et al., 2020). Relatedly, traditional masculinity is precarious; men must continually demonstrate ‘masculine’ behaviours (and avoid ‘nonmasculine’ behaviours) to maintain their social status (Vandello et al., 2008). Research suggests this precarity can lead men to perceive their distress as threatening to their masculine identity, resulting in efforts to reaffirm their masculinity via gender‐normative behaviours (see Vandello et al., 2023). These behaviours include internalising or suppressing emotional distress (Ridge et al., 2011) and engaging in risk‐taking health behaviours (e.g. substance use; Vandello et al., 2023).

Although poor mental health broadly can undermine social connectedness (Saeri et al., 2018; Yu et al., 2015), psychological distress should have stronger effects on perceived social support for men than for women due to the perceived threat it poses to traditional masculinity. Prior research indirectly supports this possibility. For instance, qualitative research suggests that men experiencing distress avoid seeking support to circumvent stigma from others (see Johnson et al., 2012; Mahalik & Dagirmanjian, 2019; Staiger et al., 2020). Avoidance of help‐seeking is especially prevalent among men who endorse traditional masculine norms (Joyce et al., 2024; van Rossum et al., 2025). Moreover, because traditional gender norms predict how men *express* their distress (see Ridge et al., 2011), men experiencing distress may (inadvertently or intentionally) signal that support is not wanted or needed and, as a result, receive less support from others. Although our study cannot test these mechanisms, these findings support our prediction that direct effects of psychological distress on perceived social support should differ by gender.

Despite the theoretical and empirical evidence discussed above, no studies to date directly examine gender differences in the *reciprocal* relationship between perceived social support and psychological distress. Indeed, most existing research on gender differences is qualitative or cross‐sectional, and the few studies that utilise longitudinal quantitative designs focus only on how social support predicts later mental health (e.g. Milner et al., 2016). Yet examining gender differences in the potentially bidirectional effects of social support and psychological distress is critical to informing health interventions. For instance, men gain fewer benefits from generalised psychosocial interventions (e.g. Krysinska et al., 2017; Wade et al., 2016) yet are more likely to die by suicide than women (World Health Organisation, 2021). Further investigating the bidirectional relationships between perceived social support and psychological distress would help identify priority areas for future interventions, including those targeting traditional gender norms.

## OVERVIEW OF THE CURRENT STUDY

The current study meets this need by examining gender differences in the longitudinal relationships between perceived social support and psychological distress. To do so, we estimate a series of multigroup random intercept cross‐lagged panel models (RI‐CLPM; Hamaker et al., 2015) using 14 annual waves of data from a large, nationwide panel sample of New Zealand adults. Unlike traditional cross‐lagged panel models, an RI‐CLPM allows us to disentangle stable, trait‐like differences between people from within‐person change over time. We thus examine whether within‐person changes from a person's typical perceptions of social support predict changes in a person's typical levels of psychological distress the following year (and vice versa). Although this approach has been utilised in prior studies of support and mental health in adolescents (Meuleman et al., 2024; Yamaguchi et al., 2023) and clinical samples (Debnar et al., 2024), our study is the first to our knowledge to test and compare these associations in women and men from a general adult population.

### Hypotheses

Prior longitudinal research suggests a bidirectional relationship between social connectedness and mental health (e.g. Debnar et al., 2024; Saeri et al., 2018; Yu et al., 2015). Accordingly, we expect a bidirectional relationship between within‐person changes in perceived social support and psychological distress. Specifically, we expect that within‐person increases in perceived social support will predict subsequent within‐person declines in psychological distress (Hypothesis 1). We also predict that within‐person increases in psychological distress will predict subsequent within‐person declines in perceived social support (Hypothesis 2). However, prior research suggests that social connectedness is a stronger predictor of mental health than vice versa (even when effects are reciprocal; see Saeri et al., 2018). Accordingly, we also test whether the within‐person effects of perceived social support on psychological distress are stronger than the within‐person effects of psychological distress on perceived social support (Hypothesis 3).

We then examine whether the associations between perceived social support and psychological distress differ between women and men. Prior work suggests gender differences in experiences of both perceived social support and psychological distress, whereby (a) perceived social support may have a stronger negative effect on psychological distress among women (e.g. Johansen et al., 2021) and (b) psychological distress may have a stronger negative effect on perceived social support among men (Nam et al., 2010; Staiger et al., 2020). Accordingly, we expect the within‐person effects of perceived social support on psychological distress will be stronger for women (compared to men; Hypothesis 4), and the within‐person effects of psychological distress on perceived social support will be stronger for men (compared to women; Hypothesis 5). Given the scarcity of longitudinal research examining our associations of interest, Hypotheses 3–5 are exploratory.

Finally, because intersections of gender and other (dis)advantaged identities influence both gender norms and well‐being (for discussion, see van Rossum et al., 2025; Wilchins, 2019), we also conduct multigroup analyses comparing women and men of different age groups, sexual identities and ethnicities. In doing so, we aim to determine the extent to which any gender differences generalise across gender groups with differing structural (dis)advantages.

## METHOD

### Sampling procedure and participants

We used data from Time 2 (2010)^1^ to Time 15 (2023) of the New Zealand Attitudes and Values Study (NZAVS)—an ongoing longitudinal panel study of New Zealand adults. Participants were initially randomly sampled from the New Zealand electoral roll, which is a compulsory list of registered voters (Time 1 [2009] *n* = 6518, response rate = 16.6%). Subsequent booster sampling occurred at Times 3 (2011), 4 (2012), 5 (2013), 8 (2016), 10 (2018), 11 (2019), 14 (2022) and 15 (2023) to diversify and increase the size of the sample. By Time 15 (2023), 76,409 unique participants had completed at least one wave of the NZAVS, with good wave‐to‐wave retention (68–86%). Informed consent was obtained from all participants. Although the NZAVS is broadly representative of New Zealand's general population, the study overrepresents women and New Zealand Europeans (Statistics New Zealand, 2024), mostly because these groups are more likely to respond to surveys. The NZAVS Open Science Framework (OSF) page provides detailed information about the sampling procedure, participant demographics, measures, attrition and retention (see https://osf.io/75snb/).

We focused on the 75,404 participants who provided partial or complete responses to our focal variables at one or more assessment occasion. Most participants completed three or more waves (66.0%; *M*_waves_ = 4.51, *SD* = 3.21; range: 1–14). Of our total participants, 62.1% were women, 36.8% were men and 1.1% were non‐binary or gender diverse. Most participants were born in New Zealand (77.3%), and the average age at Time 2 (2010) was 39.07 years (*SD* = 16.01). Concerning ethnicity, most participants identified as New Zealand European/Pākehā (89.0%), Māori (13.0%), Pasifika (3.8%) and/or Asian (6.7%; participants could report more than one ethnicity).

### Measures

Items were embedded within the larger NZAVS questionnaire. Unless otherwise specified, items were measured at every assessment occasion (i.e. from Times 2 to 15) and averaged at each assessment to represent their respective constructs. Table S1 displays the descriptive statistics and bivariate correlations between all variables included in our analyses.

#### Perceived social support

We measured perceived social support using the mean of three items from Cutrona and Russell (1987): (a) ‘There are people I can depend on to help me if I really need it’, (b) ‘There is no one I can turn to for guidance in times of stress’ (reverse‐scored) and (c) ‘I know there are people I can turn to when I need help’. Items were rated on a 1 (*strongly disagree*) to 7 (*strongly agree*) scale and displayed acceptable reliability at each assessment occasion (*ωs* = .75–.85).

#### Psychological distress

We measured psychological distress using the mean of the six‐item K6 psychological distress scale (Kessler et al., 2010). We asked participants to report how often they felt the following in the last 30 days: (a) ‘hopeless’, (b) ‘so depressed that nothing could cheer you up’, (c) ‘restless or fidgety’, (d) ‘that everything was an effort’, (e) ‘worthless’ and (f) ‘nervous’ on a 0 (*none of the time*) to 4 (*all of the time*) scale. The K6 displayed good reliability at each assessment (*ωs* = .84–.87).

#### Demographics

##### Gender

From Times 2 to 5, gender was assessed with a ‘female’ and ‘male’ binary forced‐choice question. To allow participants to describe their gender identity with their preferred terminology, we updated our gender measure from Time 6 onwards with an open‐ended question: ‘What is your gender?’. Responses were coded according to a scheme devised for the NZAVS (see Table S2 for the full coding scheme; Fraser et al., 2020). Given the small proportion of transgender, non‐binary and gender‐diverse respondents (1.1% of the entire NZAVS sample), these participants were included in our overall analyses but excluded from our multigroup analyses (where gender was dummy‐coded; 0 = woman, 1 = man). Note that very few participants explicitly reported a binary transgender identity (Fraser et al., 2020) and that many binary transgender people do not disclose that they are transgender in surveys (see Bauer et al., 2017). We therefore cannot differentiate between cisgender participants and binary transgender participants who did not disclose that they were transgender.

##### Age

We measured age using participants' date of birth. For our multigroup analyses, we separated participants into two younger (Generation Z and Millennials; 1981–2005) and older (1928–1980) age groups.

##### Sexual orientation

We assessed sexual orientation from Time 5 (2013) onwards by asking participants, ‘How would you describe your sexual orientation?’ The item wording was revised at Time 9 (2017) with prompts to minimise missing responses: ‘(e.g. heterosexual, homosexual, straight, gay, lesbian and bisexual)’. Responses were coded according to a scheme devised for the NZAVS (Greaves et al., 2017). We dummy‐coded responses based on whether participants had ever reported a sexual minority identity^2^ (0 = heterosexual; 1 = sexual minority).

##### Ethnicity

We measured ethnicity by asking participants, ‘Which ethnic group(s) do you belong to?’ Participants could select one or more of the following responses: ‘Māori, New Zealand European, Samoan, Cook Islands Māori, Tongan, Niuean, Chinese, Indian and Other (such as Dutch, Japanese and Tokelauan)’. We dummy‐coded responses based on whether participants reported a solely New Zealand European identity versus any ethnic minority group affiliation (0 = New Zealand European, 1 = ethnic minority).

### Analytic approach

We estimated a series of RI‐CLPMs (Hamaker et al., 2015) in *Mplus* v. 8.13 (Muthén & Muthén, 1998–2024). Given that covariance coverage (i.e. the proportion of complete cases on a single or pair of variables) ranged from 0.02^3^ to 0.635 (*Mean* = 0.16, *SD* = 0.13), we utilised full‐information maximum likelihood estimation (FIML) to account for missing data. FIML utilises all available data, including incomplete cases, which are inevitable in large‐scale longitudinal panel studies. Notably, FIML is more efficient and produces less biassed estimates than alternative approaches (e.g. listwise deletion), especially when data are not missing completely at random (Enders & Bandalos, 2001).

We first estimated a baseline single‐group RI‐CLPM (see Hamaker et al., 2015), testing the longitudinal within‐person associations between perceived social support and psychological distress across our entire sample (Model 1). To do so, psychological distress and perceived social support scores were decomposed into three components: grand means, between‐person components and within‐person components (see eq. 1^4^).

Equation (1a) shows the decomposing of participants' psychological distress, and Equation (1b) shows the decomposing of perceived social support scores.

(1a) $$D_{\mathrm{it}}=\mu_{t}+{\mathrm{BD}}_{i}+{\mathrm{WD}}_{\mathrm{it}},$$

(1b) $$S_{\mathrm{it}}=\pi_{t}+{\mathrm{BS}}_{i}+{\mathrm{WS}}_{\mathrm{it}}.$$

Here, $D_{\mathrm{it}}$ and $S_{\mathrm{it}}$ represent psychological distress and perceived social support scores, respectively, for person *i* at assessment *t*. The grand means for psychological distress and perceived social support are represented by $\mu_{t}$ and $\pi_{t}$, respectively. The *between‐person* (B) components are modelled as random intercepts reflecting each person's time‐invariant deviation from the grand mean (thus representing stable differences between people). The random intercepts were estimated in *Mplus* following Hamaker et al.'s (2015) approach; psychological distress and perceived support scores at each assessment were indicators of the corresponding random intercept, with means and variances of the random intercepts freely estimated but factor loadings constrained to 1. To recognise that people who tend to score higher on perceived social support also tend to score lower on psychological distress, we allowed the random intercepts to covary. Finally, the *within‐person* (W) components represent the difference between a person's observed and expected scores on psychological distress and perceived support, allowing us to examine within‐person deviations from one's ‘typical’ levels of our focal constructs. Following Hamaker et al.'s (2015) approach, the within‐person components were estimated by constraining the factor loadings at each assessment to 1 and constraining the residual variances to 0.

We then modelled the autoregressive and cross‐lagged associations between the within‐person components of psychological distress and perceived social support (see Equations 2a and 2b).

Equation (2a) shows the within‐person deviations in psychological distress, and Equation (2b) shows the within‐person deviations in perceived social support.

(2a) $${\mathrm{WD}}_{\mathrm{it}}=\alpha_{t}{\mathrm{WD}}_{i,t-1}+\beta_{t}{\mathrm{WS}}_{i,t-1}+u_{\mathrm{it}},$$

(2b) $$\;{\mathrm{WS}}_{\mathrm{it}}=\delta_{t}{\mathrm{WS}}_{i,t-1}+\gamma_{t}{\mathrm{WS}}_{i,t-1}+v_{\mathrm{it}}.$$

The autoregressive effects represent the extent to which within‐person changes in psychological distress ($\alpha_{t}$) and perceived social support ($\delta_{t}$) predict within‐person changes in the same construct at the next assessment (i.e. *inertia*). For instance, if $\alpha_{t}$ is positive, this means that an individual who experiences elevated psychological distress (compared to their typical score) at one assessment is likely to experience elevated psychological distress at the next assessment. Conversely, the cross‐lagged effects ($\beta_{t}$ and $\gamma_{t}$ for psychological distress and perceived social support, respectively) reflect the extent to which within‐person changes in one construct predict within‐person changes in a different construct at the next assessment. For instance, a negative $\beta_{t}$ indicates that within‐person increases in perceived social support predict within‐person declines in psychological distress the following year (see Hamaker et al., 2015, for further information).

After estimating the baseline model, we estimated multigroup RI‐CLPMs (Mulder & Hamaker, 2021) to examine whether the associations between these constructs differed across women and men. Specifically, we estimated a multigroup model based on (a) gender (Model 2) before estimating multigroup models based on both gender *and* (b) age (Model 3), (c) sexual minority status (Model 4) and (d) ethnic minority status (Model 5). For each multigroup model, we compared models where there were no constraints across groups to models where the within‐person estimates were constrained to equality between groups. Chi‐square difference tests were used to determine whether the addition of these constraints was tenable; if constraining the estimates significantly reduced model fit, then some of the coefficients differed across groups. Finally, we used Wald tests of parameter constraints to compare the strength of the within‐person associations within and between groups. To adjust for the number of comparisons, we set our significance threshold for Wald tests to *p* ≤ .005.

Given that we had no theoretical reason to believe that estimates would differ by the wave of assessment, we estimated stationary processes across all models. For instance, the lagged effects from Times 2 to 3 were constrained to be equal to the lagged effects from Times 3 to 4 (see Orth et al., 2021). We thus present the average within‐person associations between our focal variables. To facilitate interpretation, we rescaled perceived social support (1–7 scale) and psychological distress (0–4 scale) to a continuous 0–1 scale prior to analysis and report unstandardized estimates. *Mplus* syntax for models reported in this manuscript is available via OSF (https://osf.io/75snb/).

## RESULTS

### Measurement invariance

Before testing our hypotheses, we first tested the measurement invariance of our multi‐item psychological distress and perceived social support measures across assessments and genders. Evidence of measurement invariance is critical to ensuring the meaning of our focal constructs is stable across time and/or groups (see Milfont & Fischer, 2010; Putnick & Bornstein, 2016) and begins with estimating a measurement model with configural invariance (i.e. the same pattern of factor loadings across assessment occasions). If the model fit is acceptable (CFI ≥ .95; RMSEA ≤ .06; SRMR ≤ .08; Hu & Bentler, 1999), a more restrictive model is estimated with congeneric factor loadings constrained to equality across time and/or groups (metric invariance). If applying these constraints does not impair model fit (i.e. ∆CFI ≤ .010; see Cheung & Rensvold, 2002), equality constraints are applied to the congeneric item intercepts to estimate a more restrictive model of scalar invariance. As with comparisons between configural and metric invariance, ∆CFI ≤ .010 is used to determine whether these constraints impair model fit. At a minimum, metric invariance is required for meaningful comparisons across time and/or groups (Putnick & Bornstein, 2016).

In the present study, we first examined the longitudinal measurement invariance of psychological distress and perceived social support for women and men separately using *Mplus* v. 8.13 and FIML estimation to address missing data. Models for psychological distress and perceived social support were estimated separately to allow for precise identification of misfit (if applicable). The OSM presents the model fit summaries across measurement invariance tests. As shown in Tables S3 and S4, both psychological distress and perceived social support were scalar invariant across time for both women and men. We then estimated the cross‐time measurement invariance between groups, revealing that the multigroup models were also scalar invariant across time and genders. Accordingly, we are confident in our ability to assess both the strength of associations and mean differences between our focal constructs across time and genders.

### Model 1

The baseline RI‐CLPM fit our data well (*χ* ^2^_[357]_ = 5261.28, *p* < .001; RMSEA = 0.01 [0.013, 0.014], *p* > .999; CFI = 0.99, SRMR = 0.05; see Table 1). Table 2 displays the between‐ and within‐person estimates for this model. Turning first to the between‐person associations, Table 2 reveals that people who tended to perceive higher mean levels of social support also tended to report lower psychological distress.

**TABLE 1:** Model fit statistics across single‐ and multi‐group RI‐CLPMs of perceived social support and psychological distress.

Model	*χ* ^2^	*df*	RMSEA [90% CI]	CFI	SRMR	*Δχ* ^2^_(*df*)_	*p*‐value
Model 1: Baseline, single‐group RI‐CLPM (*N* = 75,404)	5261.28	357	0.013 [0.013, 0.014]	0.989	0.054	–	–
Multigroup RI‐CLPMs							
Model 2: Gender (*N* = 74,542)							
Free	5754.52	714	0.014 [0.013, 0.014]	0.988	0.057	–	–
Constrained	5790.58	718	0.014 [0.013, 0.014]	0.988	0.057	36.06_(4)_	<.001
Model 3: Gender and age (*N* = 74,469)							
Free	6722.79	1428	0.014 [0.014, 0.014]	0.988	0.066	–	–
Constrained	6890.85	1440	0.014 [0.014, 0.015]	0.987	0.067	168.06_(12)_	<.001
Model 4: Gender and sexual minority status (*N* = 74,542)							
Free	6603.55	1,428	0.014 [0.014, 0.014]	0.988	0.059	–	–
Constrained	6705.03	1,440	0.014 [0.014, 0.014]	0.988	0.059	101.48_(12)_	<.001
Model 5: Gender and ethnic minority status (*N* = 73,153)							
Free	6875.05	1,428	0.014 [0.014, 0.015]	0.987	0.062	–	–
Constrained	6916.90	1,440	0.014 [0.014, 0.015]	0.987	0.062	41.85_(12)_	<.001

Abbreviations: 90% CI, 90% confidence interval; CFI, comparative fit index; *df*, degrees of freedom; RMSEA, root mean square error of approximation; SRMR, standardized root mean square residual.

**TABLE 2:** Between‐ and within‐person parameters for Model 1: Single‐group RI‐CLPM (N = 75,404).

Baseline model	*b*	95% CI		SE	*t*	*p*‐value
		Lower	Upper			
Between‐person level						
Support ↔ Distress	−0.011	−0.011	−0.011	0.000	−96.005	<.001
Within‐person level						
Support_T−1_ → Support_T_	0.162	0.156	0.167	0.003	56.674	<.001
Support_T−1_ → Distress_T_	−0.029	−0.033	−0.024	0.002	−13.166	<.001
Distress_T−1_ → Support_T_	−0.047	−0.053	−0.041	0.003	−15.219	<.001
Distress_T−1_ → Distress_T_	0.184	0.179	0.190	0.003	63.890	<.001

Abbreviation: 95% CI, 95% confidence interval.

Concerning the within‐person estimates, we first report the autoregressive effects of perceived social support and psychological distress. As shown in Table 2, within‐person changes in perceived social support and psychological distress significantly predicted within‐person changes in these same constructs over time. Although autoregressive estimates reflect the stability of a given construct in a traditional CLPM, the autoregressive estimates of an RI‐CLPM reflect the extent to which within‐person changes from one's typical mean score of a given construct ‘carry over’ to a later assessment. For instance, our results reveal that a one‐unit deviation from a person's typical mean score on perceived social support predicted a 0.16‐unit departure from their levels of perceived social support at the next assessment.

To test our hypotheses, we examined the within‐person cross‐lagged relationships between perceived social support and psychological distress. Consistent with Hypothesis 1, within‐person increases in perceived social support predicted subsequent within‐person declines in psychological distress (see Table 2). Likewise, within‐person increases in psychological distress predicted within‐person declines in perceived social support (Hypothesis 2). Interestingly, the within‐person effect of psychological distress on perceived social support was stronger than the corresponding effect of perceived social support on psychological distress (*Wald*_[1]_ = 28.85, *p* < .001). Contrary to Hypothesis 3, these results suggest that within‐person changes in psychological distress more strongly predict within‐person changes in perceived social support over time than vice versa.

### Model 2: Gender

To test for potential gender differences, we first estimated a multigroup model where estimates were free to vary across women and men (χ^*2*^_[714]_ = 5754.52, *p* < .001; RMSEA = 0.01 [0.013, 0.014], *p* > .999; CFI = 0.99, SRMR = 0.06; see Table 1). We then estimated a model where these estimates were constrained to equality across groups (χ^2^_[718]_ = 5790.58, *p* < .001; RMSEA = 0.01 [0.013, 0.014], *p* > .999; CFI = 0.99, SRMR = 0.06). These constraints resulted in a significant decline in model fit (Δχ^2^_[4]_ = 36.06, *p* < .001), suggesting that some estimates differed between women and men. Accordingly, we present the results of the unconstrained model in Tables 3 and 4.

**TABLE 3:** Between‐person estimates across multigroup RI‐CLPMs.

	Women						Men					
		95% CI						95% CI				
Support ↔ Distress estimates	*b*	Lower	Upper	*SE*	*t*	*p*	*b*	Lower	Upper	*SE*	*t*	*p*
Model 2: Gender	−0.011	−0.011	−0.011	0.001	−79.914	<.001	−0.010	−0.011	−0.010	0.001	−57.508	<.001
Model 3: Gender and age												
Cohorts 1980 and older	−0.010	−0.011	−0.010	0.001	−71.651	<.001	−0.010	−0.011	−0.010	0.001	−52.920	<.001
Cohorts 1981 and younger	−0.012	−0.013	−0.011	0.001	−40.958	<.001	−0.011	−0.012	−0.010	0.001	−26.239	<.001
Model 4: Gender and sexual minority status												
Heterosexuals	−0.010	−0.011	−0.010	0.001	−75.013	<.001	−0.010	−0.010	−0.010	0.001	−54.435	<.001
Sexual minorities	−0.013	−0.014	−0.012	0.001	−25.717	<.001	−0.013	−0.015	−0.012	0.001	−17.906	<.001
Model 5: Gender and ethnic minority status												
New Zealand Europeans	−0.010	−0.010	−0.010	0.001	−68.652	<.001	−0.010	−0.010	−0.009	0.001	−50.348	<.001
Ethnic minorities	−0.014	−0.014	−0.013	0.001	−38.176	<.001	−0.012	−0.013	−0.011	0.001	−25.612	<.001

Abbreviation: 95% CI, 95% confidence interval.

**TABLE 4:** Within‐person parameter estimates across multigroup RI‐CLPMs.

	Women						Men					
		95% CI						95% CI				
Lagged path	*b*	Lower	Upper	*SE*	*t*	*p*	*b*	Lower	Upper	*SE*	*t*	*p*
Model 2: Gender												
Support_T−1_ → Support_T_	0.167	0.160	0.174	0.004	46.332	<.001	0.151	0.142	0.160	0.005	32.079	<.001
Support_T−1_ → Distress_T_	−0.027	−0.033	−0.022	0.003	−9.637	<.001	−0.032	−0.038	−0.025	0.003	−9.375	<.001
Distress_T−1_ → Support_T_	−0.038	−0.045	−0.030	0.004	−9.999	<.001	−0.064	−0.075	−0.053	0.006	−11.610	<.001
Distress_T−1_ → Distress_T_	0.175	0.168	0.182	0.004	48.119	<.001	0.201	0.191	0.210	0.005	41.887	<.001
Model 3: Gender and age												
Older (≤1980)												
Support_T−1_ → Support_T_	0.157	0.150	0.165	0.004	40.065	<.001	0.140	0.131	0.150	0.005	28.416	<.001
Support_T−1_ → Distress_T_	−0.023	−0.029	−0.017	0.003	−7.765	<.001	−0.030	−0.037	−0.024	0.003	−8.719	<.001
Distress_T−1_ → Support_T_	−0.037	−0.045	−0.029	0.004	−8.691	<.001	−0.064	−0.076	−0.052	0.006	−10.860	<.001
Distress_T−1_ → Distress_T_	0.167	0.160	0.175	0.004	42.527	<.001	0.189	0.180	0.199	0.005	37.889	<.001
Younger (≥1981)												
Support_T−1_ → Support_T_	0.221	0.203	0.239	0.009	24.158	<.001	0.232	0.203	0.261	0.015	15.854	<.001
Support_T−1_ → Distress_T_	−0.044	−0.059	−0.029	0.008	−5.722	<.001	−0.041	−0.063	−0.020	0.011	−3.714	<.001
Distress_T−1_ → Support_T_	−0.039	−0.056	−0.023	0.008	−4.723	<.001	−0.060	−0.089	−0.031	0.015	−4.030	<.001
Distress_T−1_ → Distress_T_	0.205	0.188	0.222	0.009	23.368	<.001	0.273	0.245	0.302	0.014	18.938	<.001
Model 4: Gender and sexual minority status												
Heterosexual												
Support_T−1_ → Support_T_	0.164	0.156	0.171	0.004	42.980	<.001	0.143	0.133	0.152	0.005	28.950	<.001
Support_T−1_ → Distress_T_	−0.026	−0.032	−0.021	0.003	−8.837	<.001	−0.033	−0.040	−0.026	0.004	−9.417	<.001
Distress_T−1_ → Support_T_	−0.036	−0.043	−0.028	0.004	−8.816	<.001	−0.062	−0.074	−0.051	0.006	10.681	<.001
Distress_T−1_ → Distress_T_	0.166	0.159	0.174	0.004	43.281	<.001	0.195	0.185	0.205	0.005	38.715	<.001
Sexual minority												
Support_T−1_ → Support_T_	0.189	0.167	0.211	0.011	16.934	<.001	0.211	0.181	0.241	0.015	13.719	<.001
Support_T−1_ → Distress_T_	−0.032	−0.050	−0.014	0.009	−3.524	.002	−0.019	−0.041	0.004	0.011	−1.642	.101
Distress_T−1_ → Support_T_	−0.052	−0.073	−0.030	0.011	−4.658	<.001	−0.071	−0.104	−0.038	0.017	−4.223	<.001
Distress_T−1_ → Distress_T_	0.226	0.205	0.248	0.011	20.623	<.001	0.238	0.208	0.268	0.015	15.452	<.001
Model 5: Gender and ethnic minority status												
New Zealand European												
Support_T−1_ → Support_T_	0.168	0.160	0.175	0.004	41.957	<.001	0.152	0.142	0.162	0.005	29.456	<.001
Support_T−1_ → Distress_T_	−0.025	−0.032	−0.019	0.003	−8.097	<.001	−0.030	−0.037	−0.023	0.004	−8.176	<.001
Distress_T−1_ → Support_T_	−0.040	−0.048	−0.031	0.004	−9.407	<.001	−0.064	−0.076	−0.052	0.006	10.494	<.001
Distress_T−1_ → Distress_T_	0.179	0.171	0.187	0.004	44.534	<.001	0.202	0.191	0.212	0.005	38.553	<.001
Ethnic minority												
Support_T−1_ → Support_T_	0.169	0.153	0.186	0.008	20.048	<.001	0.145	0.123	0.167	0.011	12.735	<.001
Support_T−1_ → Distress_T_	−0.035	−0.049	−0.022	0.007	−5.265	<.001	−0.036	−0.053	−0.020	0.009	−4.269	<.001
Distress_T−1_ → Support_T_	−0.031	−0.048	−0.014	0.009	−3.522	.001	−0.060	−0.086	−0.034	0.013	−4.578	<.001
Distress_T−1_ → Distress_T_	0.159	0.142	0.176	0.008	18.821	<.001	0.194	0.170	0.217	0.012	16.378	<.001

Abbreviation: 95% CI, 95% confidence interval.

Consistent with Model 1, the correlations between random intercepts revealed that women and men who perceived higher mean levels of social support also reported lower psychological distress (Table 3). Likewise, Table 4 shows the significant within‐person autoregressive effects of both perceived social support and psychological distress.

We then tested the cross‐lagged associations between perceived social support and psychological distress (see Table 4). Consistent with Model 1, within‐person increases in perceived social support predicted within‐person declines in psychological distress the following year (and vice versa). But contrary to Hypothesis 4, this association was similar in size across women and men (*Wald*_[1]_ = 0.93, *p* = .336), suggesting no gender differences in the effects of perceived social support on psychological distress.

Further analyses revealed that the within‐person effect of psychological distress on perceived social support differed across women and men (*Wald*_[1]_ = 15.10, *p* < .001). Among women, the within‐person effect of psychological distress on perceived social support was similar in size to the within‐person effect of perceived social support on psychological distress, suggesting a reciprocal relationship over time (*Wald*_[1]_ = 5.83, *p* = .016). However, for men, the within‐person effect of psychological distress on perceived social support was *stronger* than the within‐person effect of perceived social support on psychological distress (*Wald*_[1]_ = 29.66, *p* < .001). Consistent with Hypothesis 5, these results suggest that within‐person increases in psychological distress are a stronger predictor of within‐person declines in perceived social support among men (compared to women).

### Model 3: Gender and age

**FIGURE 1:**
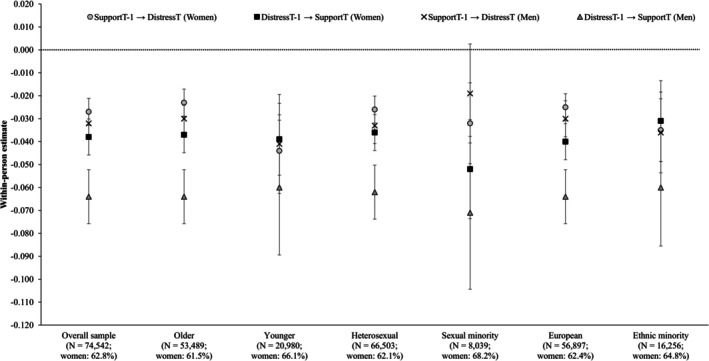
Within‐person lagged effects of perceived social support and psychological distress among women and men. *Note*: Error bars represent 95% confidence intervals. Confidence intervals that cross the dotted horizontal line (*y* = 0.000) reflect non‐significant effects (*p* > .050).

We then examined whether gender differences generalised across groups based on age (Model 3), sexual minority status (Model 4) and ethnic minority status (Model 5; see Figure 1 for comparisons of estimates across models). In Model 3, we estimated a multigroup model whereby estimates were free to vary across older women, younger women, older men and younger men (χ^2^_[1428]_ = 6722.79, *p* < .001; RMSEA = 0.01 [0.014, 0.014], *p* > .999; CFI = 0.99, SRMR = 0.07; see Table 1). We then estimated a model where the within‐person estimates were constrained to equality across the four groups (χ^2^_[1440]_ = 6890.85, *p* < .001; RMSEA = 0.01 [0.014, 0.015], *p* > .999; CFI = 0.99, SRMR = 0.07). These constraints significantly reduced model fit (Δχ^2^_[12]_ = 168.06, *p* < .001), suggesting that some estimates differed across groups.

The between‐person associations for Model 3 replicate our prior results; people perceiving higher social support also reported lower psychological distress, and this was consistent across groups (see Table 3). In terms of the autoregressive effects, within‐person deviations in psychological distress and perceived social support predicted subsequent deviations in the same constructs across all groups.

Turning to the cross‐lagged associations, within‐person changes in perceived social support negatively predicted within‐person changes in psychological distress, and this association was similar in size across the four groups (*Wald*_[3]_ = 8.74, *p* = .033; see Table 4). Similar to Model 2, however, the within‐person effects of psychological distress differed across groups (*Wald*_[3]_ = 15.42, *p* = .002). These differences were localised to older men (see Figure 1). Comparisons of estimates within groups show that the within‐person associations between perceived social support and psychological distress were negative and reciprocal over time among younger women (*Wald*_[1]_ = 0.22, *p* = .637) and younger men (*Wald*_[1]_ = 1.30, *p* = .255). Although the Wald test of parameter constraints was significant for older women (*Wald*_[1]_ = 8.21, *p* = .004), the 95% confidence intervals for the estimates overlapped, suggesting that the within‐person associations were comparable in size (see Figure 1). In contrast, the within‐person effect of psychological distress on perceived social support was stronger than the within‐person effect of perceived social support on psychological distress among older men (*Wald*_[1]_ = 28.66, *p* < .001).

### Model 4: Gender and sexual minority status

Concerning gender and sexual minority status, comparisons of the free (χ^2^_[1428]_ = 6603.55, *p* < .001; RMSEA = 0.01 [0.014, 0.014], *p* > .999; CFI = 0.99, SRMR = 0.06; see Table 1) and constrained (χ^2^_[1440]_ = 6705.03, *p* < .001; RMSEA = 0.01 [0.014, 0.014], *p* > .999; CFI = 0.99, SRMR = 0.06) models showed that estimates differed based on gender and sexual minority status (Δχ^2^_[12]_ = 101.48, *p* < .001). The between‐person component of the model revealed a negative association between perceived social support and psychological distress across groups (see Table 3). Likewise, Table 4 shows the significant within‐person autoregressive effects of perceived social support and psychological distress across groups.

We then tested for differences in the focal cross‐lagged associations. Although the cross‐lagged effects of perceived social support on psychological distress were similar in size (*Wald*_[3]_ = 3.15, *p* = .369), the cross‐lagged effect of psychological distress on perceived social support differed across groups (*Wald*_[3]_ = 16.95, *p* = .001). For women, the within‐person relationships between perceived social support and psychological distress were negative and reciprocal over time for both heterosexual (*Wald*_[1]_ = 3.97, *p* = .046) and sexual minority women (*Wald*_[1]_ = 2.31, *p* = .129; Table 4 and Figure 1). But for men, the within‐person effect of psychological distress on perceived social support was stronger than the reverse association among heterosexuals (*Wald*_[1]_ = 21.47, *p* < .001; Figure 1). Among sexual minority men, the within‐person effect of perceived social support on psychological distress was non‐significant (*b* = −0.019, *SE* = 0.011, 95% CI [−0.041, 0.004], *p* = .101), albeit similar in size to the (significant) within‐person effect of psychological distress (i.e. the 95% CIs overlapped; *Wald*_[1]_ = 8.19, *p* = .004). These results suggest that the within‐person effects of psychological distress more strongly predict reduced perceived social support than vice versa among both heterosexual and, to some extent, sexual minority men (compared to women).

### Model 5: Gender and ethnic minority status

Finally, we estimated multigroup models based on gender and ethnic minority status. Comparisons of the free (χ^2^_[1428]_ = 6875.05, *p* < .001; RMSEA = 0.01 [0.014, 0.015], *p* > .999; CFI = 0.99, SRMR = 0.06; see Table 1) and constrained (χ^2^_[1440]_ = 6916.90, *p* < .001; RMSEA = 0.01 [0.014, 0.015], *p* > .999; CFI = 0.99, SRMR = 0.06) models suggested significant differences across groups (Δχ^2^_[12]_ = 41.85, *p* < .001). As in our previous models, the between‐person associations between perceived social support and psychological distress were negative and similar across the four groups (see Table 3), and the within‐person autoregressive effects were significant for both perceived social support and psychological distress (Table 4).

In terms of cross‐lagged effects, comparisons across groups suggested no significant differences in the within‐person effects of perceived social support on psychological distress (*Wald*_[3]_ = 3.02, *p* = .389). The within‐person effects of psychological distress on social support did, however, differ across groups (*Wald*_[3]_ = 15.00, *p* = .002). The within‐person associations between perceived social support and psychological distress were negative and reciprocal among ethnic minority women (*Wald*_[1]_ = 0.20, *p* = .652) and ethnic minority men (*Wald*_[1]_ = 2.74, *p* = .098). Conversely, the within‐person effects of psychological distress on perceived social support were stronger than the reverse association among New Zealand European women (although this difference was very small; *Wald*_[1]_ = 8.71, *p* = .003) and New Zealand European men (*Wald*_[1]_ = 26.84, *p* < .001).

## DISCUSSION

Although prior research illustrates the importance of social connections for well‐being (Jetten et al., 2017; Saeri et al., 2018) and the role of traditional gender norms in social disconnection and ill‐being among men (e.g. Mostoller & Mickelson, 2024; Staiger et al., 2020), few studies explore how these processes unfold over time. We addressed this gap by testing whether the longitudinal relationships between perceived social support and psychological distress differ among women and men. Consistent with our hypotheses, results revealed that within‐person increases in perceived social support predicted subsequent declines in psychological distress 1 year later across all gender groups except sexual minority men (where this association was non‐significant). These findings extend prior longitudinal research (e.g. Saeri et al., 2018; Yu et al., 2015) by highlighting the benefits of perceived social support at the within‐person level across societal groups of intersecting gender, age, sexual and ethnic identities. Critically, however, the within‐person effects of psychological distress on perceived social support were *stronger* than the effects of perceived social support on psychological distress among men but not women. Our study thus demonstrates crucial gender differences in how psychological distress is associated with later social isolation.

Theoretically, our findings can be situated within the context of precarious manhood and gender role strain theories. Precarious manhood theory highlights how traditional masculine norms position psychological distress as incompatible with masculinity, resulting in status insecurities among men experiencing distress (see Vandello et al., 2008). To resolve these status insecurities, men are theorised to reaffirm their masculinity by engaging in gender‐normative behaviours (e.g. emotional suppression) and avoiding non‐normative behaviours (e.g. help‐seeking; Ridge et al., 2011; Vandello et al., 2008). Gender role strain theory similarly outlines how traditional masculinity constrains men's responses to distress to socially ‘acceptable’ (or prescribed) masculine behaviours (Levant & Richmond, 2016; Pleck, 1995), particularly among subgroups of men who are expected to meet high standards of traditional masculinity (e.g. White men; Levant & Wong, 2013). Together, these perspectives suggest that psychological distress inhibits emotional expression and social connectedness among men (Umberson et al., 2022; Van Rossum et al., 2025; Yousaf et al., 2015). Our findings extend this theorising, raising the possibility that psychological distress erodes men's capacity to seek and/or receive support over time, particularly among older, heterosexual and New Zealand European men. Given that older, heterosexual and White/European men are more likely to meet, endorse and be *expected* to conform to the ideals of traditional masculinity (e.g. Brassel et al., 2020; Eggenberger et al., 2022), these groups of men may be especially vulnerable to masculinity threats in response to psychological distress. Although we cannot directly examine the role of traditional masculine norms in this study, we provide (to our knowledge) the first longitudinal evidence of this phenomenon, further strengthened by our focus on intersectional groups within the general male population.

Our findings also highlight the need to target men traditionally at the margins of health research and interventions. Indeed, although women (on average) report poorer mental health than men, individual‐level interventions tend to be more effective among women (e.g. Grubbs et al., 2015), and men report lower levels of overall happiness (Blanchflower & Bryson, 2024). One way to address this discrepancy is to tailor interventions that promote positive gender norms among men, particularly those that challenge forms of masculinity that inhibit help‐seeking (King et al., 2018). Such interventions are needed at the individual level (e.g. therapeutic interventions), but also through public health messaging and community programmes to increase positive *societal* norms about men's mental health and help‐seeking (for discussion, see van Rossum et al., 2025). Future research should focus on creating and evaluating such interventions to test whether—and if so, how—they impact men's health and help‐seeking.

Although the effects of psychological distress were stronger among men, it is noteworthy that the negative effects of psychological distress on perceived social support were comparable to the effects of perceived social support among women. One possible explanation of this finding is that, irrespective of gender, acute distress fosters a self‐perpetuating help‐negation process whereby elevated distress increases one's avoidance of help and support for their symptoms, which, in turn, further elevates distress (e.g. Deane et al., 2001; Wilson, 2010; Wilson & Deane, 2010). This finding has potential implications for how and when clinicians implement ‘social cure’ interventions. Although social interventions—and shared social groups more broadly—can bolster mental health, the effects of psychological distress suggest that social interventions alone may not be sufficient to boost well‐being among those experiencing an increase in distress. It may be that successful psychological interventions to reduce psychological distress are needed *before* individuals, particularly men, can benefit from social interventions. Alternatively, interventions designed to increase social connections, address psychological distress and tackle more complex determinants of health may be necessary to promote well‐being (for discussion, see Calderón‐Larrañaga et al., 2022). We thus call for closer attention to both the general and gender‐specific effects of psychological distress on social connectedness across time and populations.

### Caveats and future directions

Although there are several strengths to the current study, particularly our use of a nationwide random sample and robust analyses that separate within‐ and between‐person effects to test our hypotheses, there are limitations worth noting. First, we focused solely on one domain of mental health (psychological distress) and one domain of social connectedness (perceived social support). Although our analyses thoroughly examined the relationship between these two constructs across different gender groups, future research should replicate our findings across different well‐being and social connectedness domains. Relatedly, studies assessing whether men actually *receive* less support in response to distress are needed to determine how broader societal norms surrounding men's help‐seeking contribute to lower social connectedness (see Eagly & Koenig, 2021). Finally, although we postulate that traditional masculine norms explain the stronger within‐person effects of psychological distress among men, the current study did not directly test this mechanism. Future research should directly assess the role of traditional gender norms and mental health stigma in undermining social support among men over time.

It is also important to note constraints on the generalisability of our findings. The current study's sample is not entirely representative of New Zealand's population (e.g. women and New Zealand Europeans were overrepresented). Fortunately, however, sample sizes across groups were sufficiently large to ensure stable parameter estimates. Coupled with the broader representativeness of the sample (Sibley, 2009–2025), this increases (but does not ensure) the generalisability of our results. Relatedly, sample size constraints in our multigroup models required the use of dummy‐coded ethnic and sexual minority groupings, which may mask important differences *within* these groups. For instance, gender differences within ethnic minority groups may emerge as a function of cultural help‐seeking and mental health norms (e.g. Abdullah & Brown, 2011). More broadly, although we explored the effects of age, sexuality and ethnicity on gender differences in our focal relationships, there are many other social identities worth exploring, including social class (Mahalik & Dagirmanjian, 2019), transgender identities (Cooper et al., 2023) and migrant status (Cabassa, 2007). In sum, our study provides a springboard for future work that investigates the effects of intersecting social identities on the relationships between mental health and social connectedness over time.

## CONCLUSION

The current study examined gender differences in the within‐person longitudinal relationships between perceived social support and psychological distress across 13 years (i.e. 2010–2023) of a nationwide longitudinal panel study. Our results suggest that both women and men receive well‐being benefits from within‐person increases in perceived social support over time. However, within‐person increases in psychological distress appear to undermine perceived social support over time, particularly among older, heterosexual and European men. These results illustrate the need to pay greater attention to how traditional gender norms may erode men's capacity to seek and perceive support in response to distress and emphasise the need for tailored social and psychological interventions. We encourage future research to further explore the role of gender in undermining social connectedness over time to ultimately redress well‐being and social connectedness disparities across groups.

## CONFLICT OF INTEREST STATEMENT

The authors declare no conflicts of interest.

## ETHICS STATEMENT

The NZAVS is reviewed every 3 years by the University of Auckland Human Participants Ethics Committee. Our current ethics approval statement for the 2021–2027 period is as follows: The New Zealand Attitudes and Values Study was approved by the University of Auckland Human Participants Ethics Committee on 26 May 2021 until 26 May 2024 and renewed on 02 May 2023 until 26 May 2027 (Reference Number: UAHPEC22576).

## Supporting information


**Table S1** Descriptive statistics and bivariate correlations between our focal variables.
**Table S2** NZAVS Gender Coding Scheme (adapted from Fraser et al., 2020).
**Table S3** Fit statistics for multigroup measurement models of psychological distress using maximum likelihood estimates.
**Table S4** Fit statistics for multigroup measurement models of perceived social support using maximum likelihood estimates.

## Data Availability

The data described in the manuscript stem from the New Zealand Attitudes and Values Study (NZAVS). Full copies of the NZAVS data files are held by all members of the NZAVS management team and advisory board. A de‐identified dataset containing the variables analysed in this manuscript is available upon request from the corresponding author or any member of the NZAVS advisory board for the purposes of replication or checking of any published study using NZAVS data. The Mplus syntax used to test all models reported in this manuscript is available on the Open Science Framework (https://osf.io/75snb/).

## ENDNOTES

1 Time 1 (2009) did not assess psychological distress.

2 We take this approach for two reasons: First, some participants initially reported a heterosexual identity and self‐disclosed their sexual minority identity at a later assessment (Lilly et al., 2023). These participants would be (inaccurately) categorised as heterosexual if we only considered their earliest reported sexual identity. Second, although our sample comprises lesbian, gay, bisexual and other sexual minority (LGB+) participants, sample sizes are too small to separate these subgroups in our analyses. Accordingly, we focus on broad differences between LGB+ and heterosexual women and men.

3 Lower covariance coverage among some variables is largely due to booster sampling between waves to diversify the sample (see Sibley, 2009–2025, for further information).

4 The equations presented here are based on those presented in Hamaker et al. (2015) and Mulder and Hamaker (2021).
