# Supplementary material for: Gender Differences in the Longitudinal Relationship Between Psychological Distress and Perceived Social Support
Source: Appl Psychol Health Well Being. 2026 Apr 29;18(3):e70148. doi: 10.1111/aphw.70148 (PMC13128988; doi:10.1111/aphw.70148)
Supplement: Supplementary file 1 — Table S1 Descriptive statistics and bivariate correlations between our focal variables. Table S2 NZAVS Gender Coding Scheme (adapted from Fraser et al., 2020). Table S3 Fit statistics for multigroup measurement models of psychological distress using maximum likelihood estimates. Table S4 Fit statistics for multigroup measurement models of perceived social support using maximum likelihood estimates. [file APHW-18-0-s001.docx]

# Gender Differences in the Longitudinal Relationship between Psychological Distress and Perceived Social Support

ONLINE SUPPLEMENTARY MATERIALS

## Table S1. *Descriptive statistics and bivariate correlations between our focal variables.*

| **Measure** | ***n*** | ***M*** | ***SD*** | **Range** | **ω** | **1** | **2** | **3** | **4** | **5** | **6** | **7** | **8** | **9** | **10** | **11** | **12** | **13** | **14** | **15** | **16** | **17** | **18** |
| --- | --- | --- | --- | --- | --- | --- | --- | --- | --- | --- | --- | --- | --- | --- | --- | --- | --- | --- | --- | --- | --- | --- | --- |
| 1. SupportT2 | 4,431 | 6.04 | 1.08 | 1–7 | .75 | -- |  |  |  |  |  |  |  |  |  |  |  |  |  |  |  |  |  |
| 1. SupportT3 | 6,880 | 6.01 | 1.11 | 1–7 | .81 | .61 | -- |  |  |  |  |  |  |  |  |  |  |  |  |  |  |  |  |
| 1. SupportT4 | 12,114 | 6.03 | 1.10 | 1–7 | .78 | .56 | .61 | -- |  |  |  |  |  |  |  |  |  |  |  |  |  |  |  |
| 1. SupportT5 | 17,687 | 6.07 | 1.10 | 1–7 | .80 | .56 | .60 | .61 | -- |  |  |  |  |  |  |  |  |  |  |  |  |  |  |
| 1. SupportT6 | 15,810 | 5.96 | 1.06 | 1–7 | .81 | .54 | .59 | .60 | .63 | -- |  |  |  |  |  |  |  |  |  |  |  |  |  |
| 1. SupportT7 | 13,935 | 6.00 | 1.06 | 1–7 | .83 | .55 | .58 | .59 | .62 | .68 | -- |  |  |  |  |  |  |  |  |  |  |  |  |
| 1. SupportT8 | 21,929 | 5.98 | 1.08 | 1–7 | .81 | .53 | .56 | .58 | .60 | .66 | .69 | -- |  |  |  |  |  |  |  |  |  |  |  |
| 1. SupportT9 | 17,062 | 5.99 | 1.08 | 1–7 | .83 | .53 | .54 | .57 | .60 | .65 | .68 | .68 | -- |  |  |  |  |  |  |  |  |  |  |
| 1. SupportT10 | 47,910 | 5.95 | 1.12 | 1–7 | .80 | .50 | .56 | .56 | .58 | .62 | .66 | .67 | .69 | -- |  |  |  |  |  |  |  |  |  |
| 1. SupportT11 | 42,653 | 5.93 | 1.15 | 1–7 | .83 | .52 | .52 | .55 | .57 | .62 | .65 | .64 | .68 | .67 | -- |  |  |  |  |  |  |  |  |
| 1. SupportT12 | 38,507 | 5.92 | 1.14 | 1–7 | .83 | .51 | .53 | .54 | .56 | .60 | .63 | .63 | .65 | .64 | .69 | -- |  |  |  |  |  |  |  |
| 1. SupportT13 | 33,934 | 5.92 | 1.17 | 1–7 | .84 | .49 | .50 | .50 | .54 | .60 | .61 | .61 | .62 | .62 | .66 | .70 | -- |  |  |  |  |  |  |
| 1. SupportT14 | 33,581 | 5.92 | 1.18 | 1–7 | .85 | .50 | .50 | .50 | .53 | .57 | .59 | .61 | .61 | .61 | .64 | .68 | .70 | -- |  |  |  |  |  |
| 1. SupportT15 | 32,639 | 5.94 | 1.17 | 1–7 | .84 | .44 | .49 | .49 | .54 | .58 | .61 | .60 | .61 | .60 | .62 | .65 | .67 | .70 | -- |  |  |  |  |
| 1. K6T2 | 4,400 | 0.84 | 0.67 | 0–4 | .84 | -.29 | -.26 | -.23 | -.27 | -.26 | -.26 | -.27 | -.27 | -.24 | -.26 | -.28 | -.30 | -.26 | -.25 | -- |  |  |  |
| 1. K6T3 | 6,791 | 0.87 | 0.70 | 0–4 | .85 | -.28 | -.32 | -.28 | -.29 | -.28 | -.29 | -.28 | -.28 | -.28 | -.29 | -.29 | -.28 | -.29 | -.27 | .66 | -- |  |  |
| 1. K6T4 | 12,028 | 0.83 | 0.67 | 0–4 | .85 | -.26 | -.27 | -.31 | -.30 | -.30 | -.29 | -.30 | -.30 | -.27 | -.28 | -.29 | -.27 | -.28 | -.27 | .65 | .67 | -- |  |
| 1. K6T5 | 17,890 | 0.86 | 0.67 | 0–4 | .84 | -.25 | -.25 | -.26 | -.32 | -.30 | -.30 | -.30 | -.30 | -.28 | -.27 | -.28 | -.28 | -.29 | -.29 | .62 | .66 | .69 | -- |
| 1. K6T6 | 15,757 | 0.83 | 0.65 | 0–4 | .84 | -.23 | -.23 | -.27 | -.28 | -.35 | -.31 | -.31 | -.32 | -.30 | -.29 | -.31 | -.30 | -.31 | -.30 | .63 | .63 | .67 | .69 |
| 1. K6T7 | 13,904 | 0.83 | 0.65 | 0–4 | .85 | -.26 | -.27 | -.27 | -.28 | -.31 | -.38 | -.33 | -.33 | -.32 | -.31 | -.31 | -.31 | -.33 | -.32 | .61 | .63 | .65 | .67 |
| 1. K6T8 | 21,849 | 0.88 | 0.68 | 0–4 | .85 | -.24 | -.23 | -.25 | -.26 | -.29 | -.31 | -.35 | -.32 | -.31 | -.30 | -.29 | -.30 | -.31 | -.30 | .57 | .62 | .64 | .65 |
| 1. K6T9 | 17,031 | 0.84 | 0.65 | 0–4 | .85 | -.23 | -.23 | -.24 | -.25 | -.29 | -.30 | -.31 | -.36 | -.32 | -.31 | -.31 | -.30 | -.31 | -.29 | .59 | .63 | .64 | .65 |
| 1. K6T10 | 47,459 | 0.90 | 0.69 | 0–4 | .85 | -.25 | -.26 | -.26 | -.26 | -.29 | -.32 | -.32 | -.33 | -.37 | -.33 | -.33 | -.32 | -.32 | -.31 | .59 | .60 | .63 | .62 |
| 1. K6T11 | 42,342 | 0.90 | 0.68 | 0–4 | .86 | -.23 | -.24 | -.25 | -.25 | -.28 | -.30 | -.29 | -.31 | -.32 | -.38 | -.33 | -.33 | -.33 | -.32 | .58 | .61 | .60 | .61 |
| 1. K6T12 | 38,252 | 0.88 | 0.68 | 0–4 | .86 | -.25 | -.24 | -.25 | -.25 | -.29 | -.31 | -.29 | -.31 | -.31 | -.34 | -.39 | -.35 | -.35 | -.34 | .58 | .59 | .61 | .60 |
| 1. K6T13 | 33,902 | 0.90 | 0.69 | 0–4 | .86 | -.20 | -.21 | -.22 | -.25 | -.27 | -.29 | -.27 | -.29 | -.29 | -.32 | -.34 | -.38 | -.35 | -.34 | .53 | .56 | .57 | .59 |
| 1. K6T14 | 33,605 | 0.93 | 0.73 | 0–4 | .87 | -.22 | -.23 | -.24 | -.23 | -.26 | -.29 | -.28 | -.30 | -.29 | -.32 | -.33 | -.34 | -.39 | -.34 | .53 | .58 | .59 | .58 |
| 1. K6T15 | 32,789 | 0.95 | 0.74 | 0–4 | .87 | -.22 | -.20 | -.25 | -.27 | -.28 | -.30 | -.27 | -.30 | -.29 | -.31 | -.32 | -.33 | -.35 | -.38 | .56 | .58 | .59 | .59 |
| 1. Gender | 74,542 | 0.37 | 0.48 | 0–1 | -- | -.17 | -.16 | -.15 | -.14 | -.16 | -.15 | -.15 | -.16 | -.14 | -.13 | -.13 | -.11 | -.11 | -.11 | -.04 | -.03 | -.04 | -.04 |
| 1. Age_(at Time 2)_ | 75,354 | 39.07 | 16.01 | 4–95^a^ | -- | -.07 | -.04 | -.06 | -.06 | -.02^*^ | -.03 | -.02^*^ | -.02^*^ | -.01 | .00 | .01 | .02^**^ | .05 | .05 | -.19 | -.28 | -.26 | -.24 |
| 1. Sexual minority | 74,542 | 0.11 | 0.31 | 0–1 | -- | .00 | -.02 | .00 | -.02^*^ | -.03 | -.03 | -.04 | -.05 | -.04 | -.05 | -.05 | -.05 | -.06 | -.06 | .09 | .10 | .07 | .11 |
| 1. Ethnic minority | 73,153 | 0.22 | 0.42 | 0–1 | -- | -.06 | -.05 | -.08 | -.07 | -.04 | -.04 | -.05 | -.05 | -.06 | -.04 | -.04 | -.04 | -.05 | -.06 | .10 | .09 | .11 | .08 |
| **Measure** | **19** | **20** | **21** | **22** | **23** | **24** | **25** | **26** | **27** | **28** | **29** | **30** | **31** | **32** |  |  |  |  |  |  |  |  |  |
| 1. K6T6 | -- |  |  |  |  |  |  |  |  |  |  |  |  |  |  |  |  |  |  |  |  |  |  |
| 1. K6T7 | .72 | -- |  |  |  |  |  |  |  |  |  |  |  |  |  |  |  |  |  |  |  |  |  |
| 1. K6T8 | .68 | .71 | -- |  |  |  |  |  |  |  |  |  |  |  |  |  |  |  |  |  |  |  |  |
| 1. K6T9 | .67 | .69 | .72 | -- |  |  |  |  |  |  |  |  |  |  |  |  |  |  |  |  |  |  |  |
| 1. K6T10 | .65 | .67 | .70 | .73 | -- |  |  |  |  |  |  |  |  |  |  |  |  |  |  |  |  |  |  |
| 1. K6T11 | .64 | .66 | .67 | .69 | .72 | -- |  |  |  |  |  |  |  |  |  |  |  |  |  |  |  |  |  |
| 1. K6T12 | .62 | .64 | .65 | .67 | .69 | .73 | -- |  |  |  |  |  |  |  |  |  |  |  |  |  |  |  |  |
| 1. K6T13 | .61 | .62 | .64 | .65 | .66 | .70 | .73 | -- |  |  |  |  |  |  |  |  |  |  |  |  |  |  |  |
| 1. K6T14 | .61 | .62 | .63 | .63 | .65 | .68 | .71 | .73 | -- |  |  |  |  |  |  |  |  |  |  |  |  |  |  |
| 1. K6T15 | .60 | .62 | .62 | .64 | .64 | .67 | .68 | .71 | .75 | -- |  |  |  |  |  |  |  |  |  |  |  |  |  |
| 1. Gender | -.04 | -.05 | -.05 | -.05 | -.04 | -.05 | -.05 | -.08 | -.08 | -.07 | -- |  |  |  |  |  |  |  |  |  |  |  |  |
| 1. Age_(at Time 2)_ | -.25 | -.24 | -.27 | -.27 | -.30 | -.31 | -.30 | -.31 | -.39 | -.40 | .07 | -- |  |  |  |  |  |  |  |  |  |  |  |
| 1. Sexual minority | .11 | .11 | .13 | .13 | .15 | .17 | .16 | .17 | .20 | .20 | -.04 | -.19 | -- |  |  |  |  |  |  |  |  |  |  |
| 1. Ethnic minority | .07 | .06 | .07 | .06 | .08 | .06 | .06 | .06 | .08 | .10 | -.02 | -.14 | .02 | -- |  |  |  |  |  |  |  |  |  |

Note. ^a^Age is measured using participants’ date of birth, defined as the age of participants at the midpoint of Time 2. This age range includes participants who turned 18 and joined the NZAVS sample at a later assessment. Values denoted in grey were non-significant (*p* > .050). ^*^*p* < .050, ^**^*p* < .010. All other values are significant, *p* < .001.

## Table S2. *NZAVS Gender Coding Scheme (adapted from Fraser et al., 2020)*

| **Level 1** | **Level 2** | **Names** | **Raw, open-ended examples from the NZAVS data** |
| --- | --- | --- | --- |
| **10** | **Female** | |  |
|  | 100 | Female | "Female," "girl," "Lady," “Straight Female,” “Woman – thank you for the open ended question,” “Mrs,” "wahine/female,” “♀,” “masculine female” |
|  | 101 | Feminine | “Feminine” |
| **20** | **Male** | |  |
|  | 200 | Male | "Male," “Identify as male,” “Mile,” “Gay male,” “MALE,” “Isn’t it obvious. Male.” |
|  | 201 | Masculine | "Masculine” |
| **30** | **Transgender** | |  |
|  | 300 | Transgender Man | “FTM,” “Male (of transgender history),” “male / transgended,” “trans male,” |
|  | 301 | Transgender Woman | “male trans mtf,” “male-to-female transsexual ???” |
|  | 302 | Transgender, not further defined | “Transgender” |
|  | 304 | Transmasculine | “Transmasculine” |
| **40** | **Non-binary/gender-diverse** | |  |
|  | 400 | Non-binary | “Non-binary”, “Non-binary transmasculine” |
|  | 401 | Genderfluid | “gender fluid”, “Gender fluid/queer,” |
|  | 402 | Primarily identifies as male, but expresses some fluidity | “Maleish” |
|  | 403 | Primarily identifies as female, but expresses some fluidity | “female (for the most part) but it complicated - gender is doing and un-doing (Butler 1990, 1993, 2004)” |
|  | 404 | Genderqueer |  |
|  | 405 | Gender diverse |  |
|  | 406 | Bigender |  |
|  | 407 | Trigender |  |
|  | 408 | Pangender |  |
|  | 409 | Polygender |  |
|  | 410 | Multigender |  |
|  | 411 | Intersex |  |
|  | 412 | Two-spirit |  |
|  | 413 | Agender |  |
|  | 414 | Nongendered |  |
|  | 415 | Genderless |  |
|  | 416 | Genderfree |  |
|  | 417 | Genderfuck |  |
|  | 418 | Graygender |  |
|  | 419 | Intergender |  |
|  | 420 | Demigender |  |
|  | 421 | Gender nonconforming |  |
|  | 422 | Gender variant |  |
|  | 423 | Third gender |  |
|  | 424 | Takatāpui |  |
|  | 425 | Fa’afafine |  |
|  | 426 | Fakaleiti |  |
|  | 427 | Androgynous |  |
| **50** | **Unsure** | |  |
|  | 500 | Unsure | "Unsure", “Not Sure” |
|  | 501 | Questioning |  |
|  | 502 | Gender Questioning |  |
| **60** | **Outside Scope** | |  |
|  | 600 | Could not be coded due to ambiguous response |  |
|  | 601 | Missing Data (just gender identity question) |  |
|  | 602 | Missing Data due to Incomplete Questionnaire Response |  |
| **41** | **Error** | |  |
|  | 4 | Error |  |
|  | 41 | Check for Error |  |

## Table S3. *Fit statistics for multigroup measurement models of psychological distress using maximum likelihood estimates.*

| **Model** | **χ2** | ***df*** | **RMSEA** | **RMSEA 90% CI** | **SRMR** | **CFI** | **ΔCFI** | **Pass** |
| --- | --- | --- | --- | --- | --- | --- | --- | --- |
| **Women** (*N* = 46,706) |  |  |  |  |  |  |  |  |
| Configural invariance | 26077.393^***^ | 2765 | 0.013 | [0.013, 0.014] | 0.055 | 0.972 | — | — |
| Metric invariance | 26454.500^***^ | 2830 | 0.013 | [0.013, 0.014] | 0.054 | 0.971 | 0.001 | Yes |
| Scalar invariance | 27571.006^***^ | 2895 | 0.014 | [0.013, 0.014] | 0.054 | 0.970 | 0.001 | Yes |
| **Men** (*N* = 27,687) |  |  |  |  |  |  |  |  |
| Configural invariance | 15813.179^***^ | 2765 | 0.013 | [0.013, 0.013] | 0.053 | 0.973 | — | — |
| Metric invariance | 16006.068^***^ | 2830 | 0.013 | [0.013, 0.013] | 0.053 | 0.973 | < .001 | Yes |
| Scalar invariance | 16522.996^***^ | 2895 | 0.013 | [0.013, 0.013] | 0.053 | 0.972 | 0.001 | Yes |
| **Multigroup** (*N* = 74,393) |  |  |  |  |  |  |  |  |
| Configural invariance | 41890.620^***^ | 5530 | 0.013 | [0.013, 0.013] | 0.054 | 0.972 | — | — |
| Metric invariance | 42531.909^***^ | 5665 | 0.013 | [0.013, 0.013] | 0.054 | 0.972 | < .001 | Yes |
| Scalar invariance | 46145.500^***^ | 5813 | 0.014 | [0.014, 0.014] | 0.054 | 0.969 | 0.003 | Yes |
|  |  |  |  |  |  |  |  |  |

*Note.* RMSEA = root-mean-square error of approximation; 90% CI = 90% conﬁdence intervals; SRMR = standardised root-mean-square residual; CFI = comparative ﬁt index. Criteria for acceptable model fit: CFI ≥ .95; RMSEA ≤ .06; SRMR ≤ .08. Criteria for acceptable model constraints (Pass = Yes): ΔCFI < .010. Conﬁgural (same factor loading patterns), metric (equal congeneric factor loadings), and scalar (equal congeneric intercepts) models were estimated sequentially.

## Table S4. *Fit statistics for multigroup measurement models of perceived social support using maximum likelihood estimates.*

| **Model** | **χ2** | ***df*** | **RMSEA** | **RMSEA 90% CI** | **SRMR** | **CFI** | **ΔCFI** | **Pass** |
| --- | --- | --- | --- | --- | --- | --- | --- | --- |
| **Women** (*N* = 46,745) |  |  |  |  |  |  |  |  |
| Configural invariance | 1192.888^***^ | 455 | 0.006 | [0.005, 0.006] | 0.020 | 0.998 | — | — |
| Metric invariance | 1380.624^***^ | 481 | 0.006 | [0.006, 0.007] | 0.021 | 0.998 | < .001 | Yes |
| Scalar invariance | 2317.998^***^ | 507 | 0.009 | [0.008, 0.009] | 0.024 | 0.995 | 0.003 | Yes |
| **Men** (*N* = 27,729) |  |  |  |  |  |  |  |  |
| Configural invariance | 913.609^***^ | 455 | 0.006 | [0.005, 0.006] | 0.019 | 0.998 | — | — |
| Metric invariance | 984.015^***^ | 481 | 0.006 | [0.006, 0.007] | 0.020 | 0.998 | < .001 | Yes |
| Scalar invariance | 1736.692^***^ | 507 | 0.009 | [0.009, 0.010] | 0.023 | 0.994 | 0.004 | Yes |
| **Multigroup** (*N* = 74,474) |  |  |  |  |  |  |  |  |
| Configural invariance | 2106.496^***^ | 910 | 0.006 | [0.006, 0.006] | 0.019 | 0.998 | — | — |
| Metric invariance | 2386.103^***^ | 964 | 0.006 | [0.006, 0.007] | 0.021 | 0.998 | < .001 | Yes |
| Scalar invariance | 4966.254^***^ | 1031 | 0.010 | [0.010, 0.010] | 0.027 | 0.993 | 0.005 | Yes |
|  |  |  |  |  |  |  |  |  |

*Note.* RMSEA = root-mean-square error of approximation; 90% CI = 90% conﬁdence intervals; SRMR = standardised root-mean-square residual; CFI = comparative ﬁt index. Criteria for acceptable model fit: CFI ≥ .95; RMSEA ≤ .06; SRMR ≤ .08. Criteria for acceptable model constraints (Pass = Yes): ΔCFI < .010. Conﬁgural (same factor loading patterns), metric (equal congeneric factor loadings), and scalar (equal congeneric intercepts) models were estimated sequentially.
